# Association between a rare novel *TP53* variant (rs78378222) and melanoma, squamous cell carcinoma of head and neck and lung cancer susceptibility in non-Hispanic Whites

**DOI:** 10.1111/jcmm.12076

**Published:** 2013-06-07

**Authors:** Xiaoxiang Guan, Li-E Wang, Zhensheng Liu, Erich M Sturgis, Qingyi Wei

**Affiliations:** a Department of Medical Oncology, Jinling Hospital, Medical School of Nanjing UniversityNanjing, China; b Department of Epidemiology, The University of Texas MD Anderson Cancer CenterHouston, TX, USA; c Department of Head and Neck Surgery, The University of Texas MD Anderson Cancer CenterHouston, TX, USA

**Keywords:** biomarker, genetic susceptibility, genotype, polymorphism

## Abstract

Recently, several studies have investigated the association between a newly reported rare functional single nucleotide polymorphism (SNP) in *TP53* (rs78378222) and cancer risk, but generated inconsistent findings. The present study further investigated this association with risk of melanoma, squamous cell carcinoma of head and neck (SCCHN) and lung cancer. Using volunteers of non-Hispanic Whites recruited for three large case–control studies, we genotyped the *TP53* rs78378222 SNP in 1329 patients with melanoma, 1096 with SCCHN, 1013 with lung cancer and 3000 cancer-free controls. Overall, we did not observe any variant homozygotes in this study population, nor significant associations between the *TP53* rs78378222AC genotype or C allele and risk for melanoma (*P* = 0.680 and 0.682 respectively) and lung cancer (*P* = 0.379 and 0.382 respectively), but a protection against SCCHN (*P* = 0.008 and 0.008 respectively), compared with the AA genotype or A allele. An additional meta-analysis including 19,423 cancer patients and 54,050 controls did not support such a risk association either. Our studies did not provide statistical evidence of an association between this rare *TP53* variant and increased risk of melanoma, nor of lung cancer, but a possible protection against SCCHN.

## Introduction

Rare variants are more likely to have a functional impact and tend to have a greater effect size than do common variants 1, 2. Thus, rare variants are likely to be a crucial genetic factor for human diseases, including cancer, and collectively they may contribute to a significant proportion of inherited susceptibility to cancer 3, 4. The *TP53* rs78378222, located in the 3′-untranslated region, is a newly identified rare SNP that is reportedly associated with risk of several cancers recently 5–7. This rare variant is within the sole polyadenylation signal of TP53, with an A-to-C change in the sequence AATAAA to AATACA. This A-to-C sequence change may result in impairing proper termination and polyadenylation of the *TP53* transcript and thus may alter cancer risk 6.

The first comprehensive and multi-institutional study identified a significant association between the *TP53* rare rs78378222C allele and risk of prostate cancer, glioma and colorectal adenoma in Caucasian populations but not with risk of colorectal cancer, breast cancer and melanoma in the same study populations 6. Subsequently, another two studies have reported that this variant allele is associated with significantly increased risk of oesophageal cancer in a Chinese population 7 and glioma in Caucasians 5. In the present study, we tested the hypothesis that the *TP53* rare rs78378222 SNP is associated with risk of aerodigestive tract cancers of the head and neck and lung, which share similar risk factors with oesophageal cancer, such as smoking, in our ongoing case–control studies of non-Hispanic Whites. In addition, we also tested the same hypothesis in our ongoing melanoma case–control study as well.

## Materials and methods

### Study population

Details of the recruitment of cases and controls have been reported elsewhere 8–10. Briefly, the non-Hispanic White volunteers with histologically diagnosed melanoma (*n* = 1329) as well as SCCHN (*n* = 1096) and lung cancer (*n* = 1013), were recruited at The University of Texas MD Anderson Cancer Center between October 1999 and October 2007; the participation rate of eligible incident cases was approximately 95% of those who were initially contacted for participation. An additional 1926 cancer-free controls for both SCCHN and melanoma studies were recruited from among hospital visitors at MD Anderson Cancer Center during the same time period, and another 1074 cancer-free controls for lung cancer from the Kelsey-Seybold Clinics, Houston's largest private multispecialty physician group. Cases and controls were frequency matched by age (±5 years), sex and ethnicity in each study. The study design, selection criteria, blood collection and DNA extraction have been described elsewhere 11.

### TP53 rs78378222 SNP genotyping

**Fig. 1:**
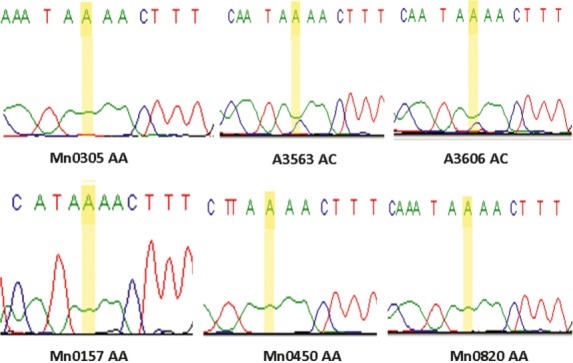
Selected sequencing samples with different genotypes of *TP53* rs78378222.

Genotyping for the *TP53* rs78378222 SNP was performed using the TaqMan assay with the Sequence Detection Software on an ABIPrism7900 (Applied Biosystems, Foster City, CA, USA), the widely used genotyping platform. Primers and probes were supplied by Applied Biosystems. For all genotypes, the assay success rate was more than 99%, and the results of repeated assays for 10% of samples were 100% concordant. Associations between variant allele/genotypes and cancer risk were estimated by computing odds ratios (ORs) and their 95% confidence intervals (CIs) from both univariate and multivariable logistic regression models with or without adjustment for age, sex, smoking and drinking status. We performed the sequencing analysis for selected samples with different genotype as shown in Figure 1.

### Mini meta-analysis

Because the association between *TP53* rs78378222 SNP and cancer risk has been tested in several case–control studies with inconsistent results, we summarized published case–control association studies of TP53 variant rs78378222 and cancer risk, then conducted a meta-analysis for all published studies of Caucasians (excluded one Chinese study) 7, using the published data sets in addition to our data. All statistical methods were described elsewhere for association analysis 11 and for meta-analysis 12.

### Statistical analysis

Differences in selected demographic variables, smoking status and alcohol use between cases and controls were evaluated by the chi-square test. The associations between *TP53* rs78378222 SNP and cancer risk were estimated by computing ORs and their 95% CIs from both univariate and multivariate logistic regression models in case–control analysis. All tests were two-sided, and *P* < 0.05 was considered statistically significant. All statistical analyses were performed with SAS software (version 9.1.3; SAS Institute Inc., Cary, NC, USA), unless stated otherwise.

## Results

### Frequency distributions of age, sex, and TP53 rs78378222 between cancer patients and cancer-free controls

As shown in Table 1, there were no significant differences in distributions of age between controls and cases of SCCHN (*P* = 0.722), lung cancer (*P* = 0.537), melanoma (*P* = 0.641) and all cases combined (*P* = 0.790), nor in distributions of sex for SCCHN (*P* = 0.537), lung cancer (*P* = 0.521), melanoma (*P* = 0.247). Among the combined cases, 2073 (60.2%) were male, whereas 1686 (55.9%) were male in all the controls. The genotype frequencies of the TP53 rs78378222 SNP in both cases and controls were in agreement with the Hardy–Weinberg equilibrium.

**Table 1:** Frequency distributions of age and sex between cancer patients and cancer-free controls

Variables	SCCHN*		*P*†	Lung cancer		*P*†	Melanoma		*P*†	Combined‡		*P*†
			
	Cases	Controls		Cases	Controls		Cases	Controls		Cases	Controls	
	*n* (%)	*n* (%)		*n* (%)	*n* (%)		*n* (%)	*n* (%)		*n* (%)	*n* (%)	
	1097	1086		1014	1076		1331	1311		3442	3015‡	
Age (y)												
Range	18–90	20–87	0.722	21–94	28–92	0.567	18–84	18–87	0.641	18–94	18–92	0.790
≤50	300 (27.4)	311 (28.6)		210 (20.7)	207 (19.2)		611 (45.9)	585 (44.6)		1121 (32.6)	988 (32.8)	
51–60	391 (35.6)	389 (35.8)		217 (21.4)	247 (23.0)		385 (28.9)	401 (30.6)		993 (28.8)	847 (28.1)	
>60	406 (37.0)	386 (35.6)		587 (57.9)	622 (57.8)		335 (25.2)	325 (24.8)		1328 (38.6)	1180 (39.1)	
Sex												
Male	826 (75.3)	830 (76.4)	0.537	457 (45.1)	500 (46.5)	0.521	790 (59.4)	749 (57.1)	0.247	2073 (60.2)	1686 (55.9)	<0.01
Female	271 (24.7)	256 (23.6)		557 (54.9)	576 (53.5)		541 (40.6)	562 (42.9)		1369 (39.8)	1329 (44.1)	

* SCCHN: squamous cell carcinoma of head and neck.

† Chi-Square test.

‡ Four hundred and fifty-eight controls overlapped between HN and melanoma studies.

### Association between a rare novel TP53 variant (rs78378222) and cancer susceptibility in non-Hispanic Whites

As shown in Table 2, TP53 rs78378222 was not associated with overall cancer risk (OR = 0.80; 95% CI = 0.58–1.11) in all cases combined, nor for risk of lung cancer (OR = 0.85; 95% CI = 0.48–1.53) and melanoma (OR = 1.10; 95%; CI = 0.68–1.77). However, this variant was protective for SCCHN risk (OR = 0.41; 95% CI = 0.21–0.79), a finding apparently not to be consistent with those reported in the literature.

**Table 2:** Association between *TP53* rs78378222 SNP and cancer risk*

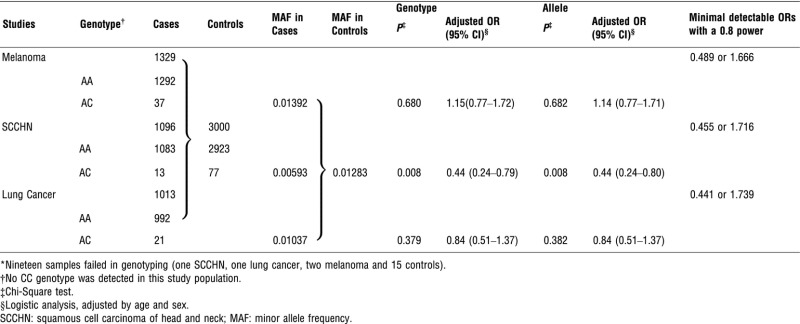

### Mini meta-analysis on the association between the TP53 rs78378222C allele and overall cancer risk

**Fig. 2:**
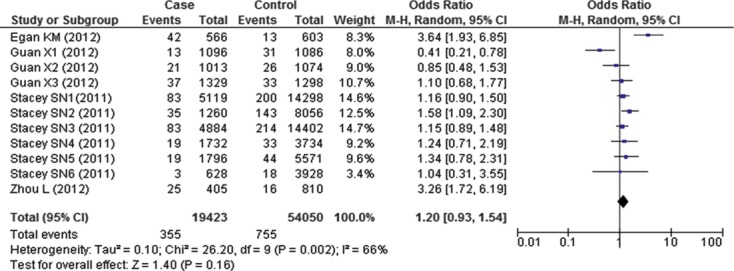
Meta-analysis of association between TP53 variant rs78378222 and cancer risk. OR and 95% CI were calculated using an allelic model for rs2736098. Guan X1-3 represented for SCCHN, lung cancer and melanoma studies, respectively; Stacey SN1-5 represented studies for prostate, glioma, colorectal, breast, melanoma and basal cell carcinoma cancers respectively.

We then conducted a mini meta-analysis of available data from published studies on the association between TP53 variant rs78378222 and cancer risk. Only three studies of Caucasian populations in addition to our data were included 5, 6, a Chinese study with small sample data set was excluded 7. As a result, we found that, overall, the pooled data showed that TP53 rare variant was not significantly associated with cancer risk (OR = 1.20; 95% CI = 0.93–1.54; Fig. 2).

## Discussion

In this study of an association between the newly reported *TP53* rs78378222 SNP (or C allele) and risk of melanoma as well as SCCHN and lung cancer in a non-Hispanic White population, we did not find a statistical evidence of an association between this rare *TP53* SNP and increased risk of melanoma, nor for lung cancer. However, we did observe a protection against SCCHN risk, although this finding needs to be further validated in large studies.

In previously published studies, the newly reported *TP53* variant rs78378222C allele was found to be associated with significantly increased risk of prostate cancer, glioma and colorectal adenoma, but not of colorectal cancer in Caucasian populations 6, and subsequent studies also confirmed the association for glioma in 566 cases and 603 controls in Caucasians 5 and reported an additional association with oesophageal cancer of the aerodigestive tract in 405 cases and 810 controls of a Chinese population 7.

In the present study, we intended to replicate the reported associations for melanoma and oesophageal cancer. A null association with melanoma risk is like to be true, given the findings from our meta-analysis. However, our findings of no association with lung cancer but a protection against SCCHN are quite different from that of oesophageal cancer. Obviously, such an association is far from established, which may be explained by several possibilities. First, numerous mechanisms involved in dysfunction of p53 and its molecular network 13, 14, which are likely to have a more functional impact on carcinogenesis than this rare variant alone. Second, a spectrum of *TP53* somatic mutations has likely participated in tumour promotion and progression that may be different in cancers of different origins 15. Third, it is likely that the very same variant in the 3′-untranslated region of *TP53* has a different role in somatic cells of different target tissues and in concert with different carcinogens exposed. Finally, there may be ethnic and geographic differences in the aetiology of different cancers, aerodigestive cancers in particular, in addition to cancer risk associated with this variant.

In our post-hoc power calculation (Table 2), we found that our sample size could detect minimal ORs of, 0.489 or 1.666 for melanoma, 0.441 or 1.739 for lung cancer and 0.455 or 1.716 of SCCHN with a statistic power of 0.8, suggesting that our findings could be by chance, although the reported positive ORs ranged from 1.60 to 3.54 5–7. In conclusion, our results suggest that the novel but rare variant of *TP53* is not associated with risk of melanoma, which is similar to the finding of a meta-analysis including 19,423 cancer patients and 54,050 controls; nor a risk association for lung cancer but a possible protection against SCCHN. However, larger and well-designed prospective studies are warranted to confirm our findings.
